# A Radiation-Free Approach Based on the Whole-Body MRI Has Shown a High Level of Accuracy in the Follow-Up of Lymphoma Patients—A Single Center Retrospective Study

**DOI:** 10.3390/jcm13133637

**Published:** 2024-06-21

**Authors:** Antonio Frolli, Sivlia Varvello, Annalisa Balbo Mussetto, Daniela Gottardi, Martina Bullo, Silvia Marini, Giuseppe Saglio, Stefano Cirillo, Daniela Cilloni, Guido Eugenio Parvis

**Affiliations:** 1 Department of Clinical and Biological Sciences, University of Turin, 10124 Turin, Italy; martina.bullo@unito.it (M.B.); silvia.marini@unito.it (S.M.); giuseppe.saglio@unito.it (G.S.); daniela.cilloni@unito.it (D.C.); 2 Division of Hematology, Mauriziano Hospital, 10128 Turin, Italydgottardi@mauriziano.it (D.G.); gparvis@mauriziano.it (G.E.P.); 3 Division of Radiology, Mauriziano Hospital, 10128 Turin, Italy; annalisa.balbomussetto@gmail.com (A.B.M.); scirillo@mauriziano.it (S.C.)

**Keywords:** lymphoma follow-up, whole-body magnetic resonance, radiation-free approach, follicular lymphoma

## Abstract

**Background:** Recurrence, even after years from the last treatment, characterizes lymphoproliferative disorders. Therefore, patients in complete remission from the disease should be followed up with periodic clinical checks. There is not a consensus on the role of imaging for this aim, because the radiological techniques used at the time of diagnosis expose patients to a risk of ionizing radiation damage. Whole-body magnetic resonance imaging with diffusion-weighted imaging (WB-MRI-DWI) has given similar results to gold standard techniques in detecting lymphoma in the involved sites without ionizing radiation. In this retrospective real-life study, we aimed to assess the accuracy of WB-MRI-DWI during follow-ups of lymphoma patients in terms of sensitivity, specificity, positive predictive value (PPV), and negative predictive value (NPV). **Methods**: Lymphoma patients who were subject to at least one WB-MRI-DWI during follow-up between February 2010 and February 2022 were enrolled. **Results**: Based on our investigation, the calculated sensitivity of WB-MRI-DWI was 100% (95% CI: 99.4–100.0), the specificity was 98.6% (95% CI: 97.4–99.3), PPV was 79% (95% CI: 75.9–81.9), and NPV was 100% (95% CI: 99.4–100.0). **Conclusions**: Despite the possibility of poor patient compliance and the identification of false positives, WB-MRI-DWI examination demonstrated an excellent sensitivity in ruling out the disease relapse.

## 1. Introduction

Lymphoproliferative diseases are a heterogeneous group of malignant neoplasia that arise from a clonal proliferation of lymphocytes. The prognosis of patients affected by lymphoid malignancies has considerably improved during the last decades, because of both more effective treatments and improved diagnostic accuracy [1]. Nevertheless, recurrence, even after years from the last treatment, has been observed in many lymphoproliferative disorders. Therefore, it is mandatory to start a follow-up (FU) for all patients in complete remission at the end of therapy.

At the present time, multidetector computed tomography with intravenous iodinated contrast enhancement (CE-CT) and 2-[18F]-fluoro-2-deoxy-D-glucose positron-emission tomography (18F-FDG-PET) are considered the gold standards for the staging of lymphoproliferative disorders at diagnosis, for evaluating the response at the end of therapy, and for the identification of recurrence of the disease [2]. Although the combined use of CE-CT and 18F-FDG PET-CT plays a crucial role in the management of patients affected by lymphoma, both examinations imply a considerable dose of ionizing radiation, increasing the risk of secondary malignances [3,4]. Additionally, the optimal number and timing of scans during follow-up to improve survival has not been established. While clinical evaluation and blood tests are crucial in monitoring any type of lymphoproliferative disorder, imaging involving ionized radiation are not recommended during FU, despite being commonly used in daily practice in relation to some types of lymphoma [5,6,7,8,9]. Therefore, radiation-free diagnostic tools need to be considered to permit an early identification of disease relapse. Among the various instrumental options, ultrasonography (US) has assumed an increasingly prominent role in the follow-up surveillance because it is a low-cost method with no radiation exposure [9]. At the same time, it has some important limitations: it has low accuracy when it comes to evaluating intrathoracic organs and bone localizations, it is also an operator-dependent method, and it can be influenced by the patient’s habitus. Finally, US assessment is only morphological and not functional [10,11].

Whole-body magnetic resonance imaging (WB-MRI) is associated with a degree of accuracy with respect to defining morphological details of disease extension similar to that associated with CE-CT and 18F-FDG PET-CT, but it is free from any ionizing radiation [12,13]. Moreover, the development of diffusion-weighted imaging (DWI) has transformed conventional WB-MRI into a morphological and functional imaging technique: the lymphomatous tissue is characterized by high cellularity and a high nucleus/cytoplasm ratio, leading to a significant reduction in the diffusivity of water molecules that results in an altered signaling by the involved tissues which allows for the identification of disease position [14]. Some study demonstrated an excellent agreement between WB-MRI with DWI (WB-MRI-DWI) and 18F-FDG-PET-CT with respect to the detection of both nodal and extra nodal locations [15,16,17]. WB-MRI-DWI seems superior to both 18F-FDG-PET-CT and CE-CT in relation to indolent lymphomas with variable FDG avidity [14]. Therefore, WB-MRI-DWI may offer an alternative diagnostic tool to traditional radiation-based procedures.

The use of WB-MRI-DWI in lymphoma detection is currently studied relative to staging (at diagnosis and at the end of treatment) and in bone marrow involvement [17,18,19]. However, there are no published data regarding instrumental surveillance during FU of these patients.

In this retrospective real-life study, we aimed to assess the accuracy of WB-MRI-DWI during FU of patients affected by lymphoma in terms of sensitivity, specificity, positive predictive value (PPV), and negative predictive value (NPV).

## 2. Materials and Methods

Patients with a diagnosed lymphoma who were subject to at least one WB-MRI-DWI during FU between February 2010 and February 2022 were enrolled. Data were collected from WB-MRI scans locally performed either in addition to or instead of the more widely used imaging techniques (CE-CT, 18F-FDG PET-CT, and US).

All WB-MRI-DWI examinations were performed on a 1.5 T MRI system (Achieva e Ingenia, Philips Medical System, Best, The Netherlands). Examination techniques and image interpretation, as per local policy, have already been published by Balbo-Mussetto et al. in a similar cohort of newly diagnosed lymphoma patients [13]. Both CE-CT and WB-MRI-DWI images were evaluated by two expert radiologists blinded to the other technique findings. Any initial disagreement between the two observers about the image findings was resolved when a consensus was reached. All images were subjected to systematic per-lesion and per-patient evaluations. Per-lesion assessments were based on the presence of disease in each of the following 25 nodal sites: higher and lower right lateral cervical lymph nodes, higher and lower left later cervical lymph nodes, right supraclavicular lymph nodes, left supraclavicular lymph nodes, right axillary lymph nodes, left axillary lymph nodes, right upper mediastinal lymph nodes, left upper mediastinal lymph nodes, anterior superior mediastinal lymph nodes, right pulmonary hilar lymph nodes, left pulmonary hilar lymph nodes, right lower mediastinal lymph nodes, anterior lower mediastinal lymph nodes, left lower mediastinal lymph nodes, right upper abdominal and mesenteric lymph nodes, left upper abdominal and splenic lymph nodes, right lower abdominal lymph nodes, left lower abdominal lymph nodes, right common iliac lymph nodes, left common iliac lymph nodes, pelvic and pre-sacral lymph nodes, right inguinal lymph nodes, and left inguinal lymph nodes. Per-lesion assessments were also based on the presence of disease in each of the following extra nodal sites: lung, pleura, pericardium, chest wall, liver, spleen, kidney, stomach, bowel, pancreas, and BM [20]. On morphological images, lymph nodes were considered to be pathological if (1) the short axis diameter was greater than 10 mm in the neck and mediastinum stations, (2) the short axis diameter exceeded 15 mm in the abdomen, (3) the abnormal nodes were observed in a region where no lymph nodes would normally be detected, and (4) the suspected lesions were necrotic, regardless of diameter. All DWI images were qualitatively interpreted in association with apparent diffusion coefficient (ADC) maps. ADC measurements were not used for tissue characterization because the ADC cut-off values reported for differentiating malignant and non-malignant lesions have been shown to be inconsistent [21]. All lymph nodes with signal intensity higher than that of the spinal cord were considered to be positive for lymphomatous involvement, whereas, in the extra nodal sites, every area of abnormal signal intensity relative to the surrounding tissue was considered to be pathological. For tissues with normally impeded diffusion (i.e., brain, spinal cord, peripheral nerves, salivary glands, gallbladder, small intestine and colonic contents, tonsils, spleen, kidneys, adrenal glands, prostate, testes, penis, endometrium, ovaries, and BM), any focally increased signal intensity was considered to be positive for tumor involvement.

Due to the retrospective nature of the study, no direct comparison between WB-MRI and other imaging techniques was carried out. Results from WB-MRI-DWI were compared to clinical information (observation, imaging, and histological data) in order to define cases of true and false positivity or negativity. A WB-MRI-DWI scan was considered to be a true negative if no signs of relapse (clinical or based on another imaging techniques or biopsy) occurred in the three months after the scans. Subsequently, sensitivity, specificity, PPV, and NPV were calculated.

All patients provided written informed consent to the WB-MRI procedure. The retrospective analysis was approved by the Local Ethics Committee.

## 3. Results

### 3.1. Patients Characteristics

Between February 2010 and February 2022, 191 patients with a histologically confirmed diagnosis of lymphoma underwent at least one WB-MRI-DWI during the follow-up period. The main patients’ characteristics are shown in Table 1. Briefly, most of the enrolled patients were affected by Hodgkin lymphomas (HL—*n* = 75 [39.3%]), followed by diffuse large B-cell lymphomas (DLBCL—*n* = 47 [24.6%]), and follicular lymphomas (FL—*n* = 33 [17%]). The median age at the start of FU was 47 years (range 16–80). The median duration of FU was 54 months. A total of 691 WB-MRI analysis were performed. Each patient was subjected to a median of three WB-MRI-DWI.

### 3.2. WB-MRI-DWI Accuracy

Out of the total 156 patients enrolled, 33 (17.2%) were associated with at least one WB-MRI-DWI positive examination for either disease progression or relapse, while the remaining 158 (82.7%) were judged to be negative. Among the positive ones, 26 (78.7%) were confirmed to be true positive (TP) events. The remaining seven patients (21.2%) turned out to be false positives (FP) upon further histological investigation. Interestingly, no false negatives (FN) were recorded among those patients with a negative WB-MRI-DWI image for lymphoma progression or relapse. Out of the 691 WB-MRI scans analyzed, 43 (6.3%) were judged to be positive for either disease progression or relapse, while the remaining 648 (93.7%) were judged to be negative. Among the positive ones, 34 (79.0% of the positive cases—95% CI: 66.8–91.2) were confirmed to be true positive (TP) events and correlated with clinical relapse. The remaining nine scans (21% of the positive cases—95% CI: 8.8–33.2) turned out to be false positives (FP) upon further investigation. Interestingly, no false negatives (FN—0% of total—95% CI: 0.0–1.9) were recorded among those patients with a negative WB-MRI-DWI image for lymphoma progression or relapse (all true negatives, TN).

**Figure 1:**
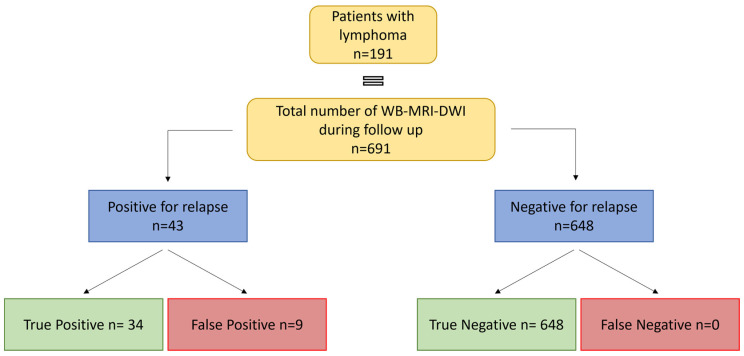
Structure of the study.

Based on our assessment of the patients, the calculated sensitivity of WB-MRI-DWI in lymphoma surveillance was 100% (95% CI: 90.2–100.0), while specificity was 95.7% (95% CI: 92.6–98.8). The positive predictive value (PPV) was 79% (95% CI: 66.8–91.2) and the negative predictive value (NPV) was 100% (95% CI: 97.5–100.0). A study design is reported in Figure 1

### 3.3. Positive WB-MRI-DWI

As mentioned above, out of 43 (6.3% of the total) positive WB-MRI-DWI scans for lymphoma progression or relapse, 34 (79%) were TP and nine (21%) were FP. These scans were derived from 33 patients. The main characteristics of these patients are shown in Table 2. Briefly, the histological subtypes included 14 HL, nine DLBCL, seven FL, one MCL, one MZL, and one PTCL

Among the 27 patients who relapsed, 19 were completely asymptomatic and the WB-MRI-DWI allowed an early identification of the clinical event as demonstrated in the following instrumental analysis. In the remaining eight cases (27.2%), WB-MRI-DWI confirmed a previous clinical or radiological suspicion.

Among the seven FP lymphoma cases, the histological subtypes included five HD and two DLBCL. None of these patients showed symptoms attributable to clinical relapse and WB-MRI-DWI was the only imaging technique performed during FU.

**Figure 2:**
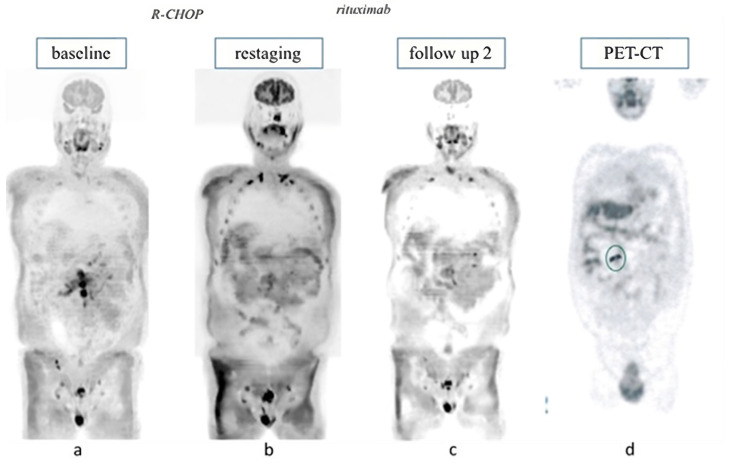
A case of follicular lymphoma with positive WB-MRI-DWI analysis at diagnosis (**a**) and negative scans at restaging after achieving complete relapse with R-CHOP treatment (**b**) is reported. During rituximab maintenance, WB-MRI-DWI (**c**) and PET (**d**) scans were done for high clinical suspicion of relapse. WB-MRI-DWI analysis ruled out the relapse, while PET was judged to be positive (the circle in the image (**d**) represents the PET positive lesion). A biopsy was performed on PET suspected lesions and lymphoma presence was excluded.

The confirmation of false positivity was obtained following further diagnostic procedures. Four patients were studied with 18F-FDG PET-CT after WB-MRI-DWI positivity: in only one case, the procedure resulted negative, thus confuting the relapse suspicion, while, in the other three cases, the results were null (Figure 2). The four patients underwent a targeted biopsy of the suspected lesion, all of which came back negative for disease presence. In two cases, the confirmation of the absence of the lymphoma was obtained with a stringent imaging follow-up with other RM or US.

### 3.4. Negative WB-MRI-DWI

**Figure 3:**
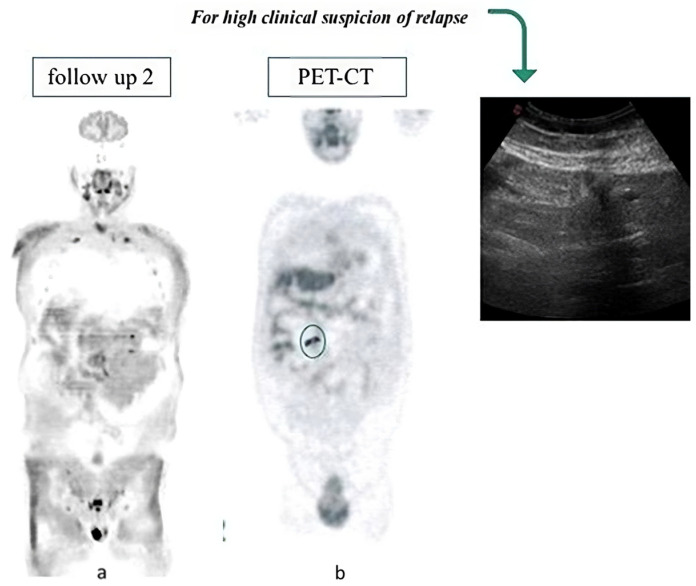
Negative WB-MRI-DWI (**a**) with positive PET image (**b**) (the circle in the image (**b**) represents the PET positive lesion). The absence of disease was confirmed by a following US scan and clinical monitoring.

A total of 648/691 scans (93.7% of total) presented one or more negative WB-MRI-DWI. As expected, most of the histological subtypes in this subgroup were “aggressive lymphomas”: 48 [40.3%] HL and 33 [27.3%] DLBCL, followed by 17 (14.3%) FL. Of note, two cases were associated with a negative WB-MRI-DWI scan despite 18F-FDG PET-CT-derived positivity (Figure 3). However, clinical progressions were not confirmed by further investigations; thus, no FN were recorded and WB-MRI-DWI scans proved to be fundamental for demonstrating the complete remission status.

## 4. Discussion

This retrospective study allowed us to evaluate the role of WB-MRI in FU of lymphoproliferative disorders in our Centre.

Out of the 191 patients analyzed, the main lymphoma types included HL, DLBCL, and FL. Apart from epidemiological reasons, this selection bias was probably related to the urgent medical need of a useful imaging technique with high diagnostic value and low radiation exposure, especially in this subgroup of patients. Indeed, the expected low median age (47, range: 16–80) of our study population, with a high presence of young HL patients (median age 32 y range: 16–80), confirms the mandatory requirement of a highly sensitive technique associated with low toxicity [22,23].

ESMO and NCCN guidelines consider imaging optional during follow-up periods of lymphoma patients, with techniques such as CE-CT and 18F-FDG PET-CT related to radiation exposure risk, especially in young patients [5,6,7,8,9]. In our assessment, WB-MRI represented an additional imaging tool to the gold standard examinations. However, we hypothesized that the high reliability of the technique would justify the cost of the examination, both in terms of economic costs and patient’s effort.

The absence of FN cases and the consequent 100% sensitivity shown by WB-MRI-DWI in our lymphoma cohort are the main findings of our analysis. In the same way, the NPV was 100%. These results are particularly interesting considering the young age of the population and the need for a diagnostic instrumental analysis which can allow exclusion, with reasonable certainty, of the presence of the disease.

On the other hand, the specificity of the technique was found to be 95.7%, with a PPV of 79.0%, indicating that a false positive rate of 20% affected all positive results.

Only the FP cases (seven patients in total) did not benefit from WB-MRI-DWI during surveillance. As a matter of fact, those patients underwent further investigations (mainly biopsy or 18 FDG PET-CT) to confirm the absence of a lymphoproliferative disease. However, we thought it would be better to offer our patients a slightly overestimating technique rather than a higher risk of FN cases, not correctly identifying all events of disease relapse.

Usually, the relapse occurs early after first-line therapy in cases of aggressive lymphoma, and WB-MRI-DWI could be a useful tool during the first years of FU. On the other hand, costs and patient tolerability remain major limitations of WB-MRI-DWI.

Finally, although the use of WB-MRI-DWI is nowadays more and more suggested in numerous trials at high risk of developing asymptomatic cancer in the patient subjects, its use in lymphoma FU is only one of the possible courses of action in oncology surveillance [24].

Considering the histological subtypes, the use of WB-MRI-DWI FU of FL has already been supported by some authors [18,25]. Because of the pain-free nature of this type of lymphoma, it is important to identify a reliable and accurate imaging technique to promptly identify disease progression while keeping radiation exposure to the lowest level possible. Notably, in our study, out of the 33 FL enrolled, both NPV and PPV were found to be 100%, with no FN nor FP, thereby confirming the validity of using WB-MRI-DWI in the FU of these patients.

In cases of aggressive lymphomas, a prompt start of treatment can impact significantly on the history of the disease and, therefore, on survival. In the same way, an early diagnosis in asymptomatic patients could extend their life expectancy. WB-MRI-DWI, by identifying sub-clinical diseases in asymptomatic patients, could meet this need and permit the beginning of an early treatment.

The retrospective nature of this study does not allow for a direct comparison between WB-MRI-DWI and other radiological techniques such as 18 FDG PET-CT or CE-CT. Data obtained from a meta-analysis and literature review have shown that the use of 18 FDG PET-CT in the follow-up of lymphoma-affected patients suffers from a high number of false positives (42.9%) [26]. A direct comparison between 18 FDG PET-CT and WB-DWI-MRI in the field of disease staging at diagnosis is available in the literature. Both methods showed comparable sensitivity and specificity values with respect to the detection of lymph node involvement in the disease (sensitivity and specificity of 98.2% and 99% for WB-DWI-MRI, respectively, and sensitivity and specificity of 99.4% and 100% for 18 FDG PET-CT, respectively), but, in detecting splenic occurrences, 18 FDG PET-CT was found to exhibit better accuracy (accuracy of 18 FDG PET-CT was 100%, accuracy of WB-DWI-MRI was 83%) while WB-DWI-RM exhibited better accuracy with respect to the identification of bone marrow events (accuracy of 18 FDG PET-CT was 79%, accuracy for WB-DWI-MRI was 93%) [16].

In the settings of disease staging, the overlap in the methods was demonstrated in relation to aggressive forms of lymphomas (accuracy 98% for both methods), whereas, in pain-free lymphomas, WB-DWI-MRI seemed to provide better results (accuracy of 18 FDG PET-CT and WB-DWI-MRI was 87% and 98%, respectively) [15].

In conclusion, WB-MRI-DWI appears to be a very effective technique to confirm the disease remission status in the follow-up of lymphoma patients. The limitation of this study consisted of its retrospective nature and the need to define, through prospective studies, the timing for patients’ evaluation during the follow-up.

## Figures and Tables

**Table 1:** Baseline characteristics of patients. HL = Hodgkin Lymphoma, DBLCL = diffuse large B-cell lymphoma, FL = follicular lymphoma, MZL = marginal zone lymphoma, PMBCL = primary mediastinal large B-cell lymphoma, MCL = mantle cell lymphoma, BL = Burkitt lymphoma, PTCL = peripheral T-cell lymphoma, PTLD = post-transplant lymphoproliferative disorder, ALCL = anaplastic large cell lymphoma, ATCL = angioimmunoblastic T-cell lymphoma, LPL = lymphoplasmacytic lymphoma, PBL = plasmablastic lymphoma.

Number of Patients	191
Median age, years (range)	47 (16–80)
Male sex, *n* (%)	97 (50.8)
Histology, *n* (%)	
HL	75 (39.3)
DLBCL	47 (24.6)
FL	33 (17.3)
MZL	10 (5.2)
PMBCL	7 (3.7)
MCL	6 (3.1)
BL	3 (1.6)
PTCL	3 (1.6)
PTLD	2 (1.0)
ALCL	2 (1.0)
ATCL	1 (0.5)
LPL	1 (0.5)
PBL	1 (0.5)
Median follow up, months (range)	54 (3–140)
Median WB-MRI-DWI for patient (range)	3 (1–13)

**Table 2:** Characteristics of patients with positive WB-MRI-DWI scans for relapse.

Number of Patients	33
Median age, years (range)	51 (18–80)
Male sex, *n* (%)	20 (60.6)
Histology, *n* (%)	
HL	14 (42.5)
DLBCL	9 (27.3)
FL	7 (21.2)
MZL	1 (3.0)
MCL	1 (3.0)
PTCL	1 (3.0)
Median follow-up, months (range)	57 (3–125)
Median WB-MRI-DWI per patient (range)	4 (1–13)

## Data Availability

Data are contained within the article.
